# Southern Medical College, Atlanta

**Published:** 1883-10-20

**Authors:** 


### *SOUTHERN MEDICAL COLLEGE, ATLANTA.*

The above Institution opens with an increased class the present session, and encouraging prospects for the future.

The school is well officered and equipped in all departments. The clinical advantages are excellent, and there is a hospital connected with the Institution. Practical Anatomy is here most thoroughly taught, in which the students have every facility, including dissections, plates, models, stereopticon illustrations, etc., and also Chemistry, Physiology, Hygiene, Obstetrics, Surgery and the special departments of the Eye, Ear and Throat, Dermatology, Dentistry, etc.

The Institution well deserves the patronage and encouragement of the Profession everywhere.

